# Data related to the nanoscale structural and compositional evolution in resistance change memories

**DOI:** 10.1016/j.dib.2018.09.087

**Published:** 2018-10-03

**Authors:** Taimur Ahmed, Sumeet Walia, Edwin L.H. Mayes, Rajesh Ramanathan, Paul Guagliardo, Vipul Bansal, Madhu Bhaskaran, J. Joshua Yang, Sharath Sriram

**Affiliations:** a Functional Materials and Microsystems Research Group and Micro Nano Research Facility, RMIT University, Melbourne, VIC 3001, Australia; b RMIT Microscopy and Microanalysis Facility, RMIT University, Melbourne, VIC 3001, Australia; c Ian Potter NanoBioSensing Facility, NanoBiotechnology Research Laboratory, School of Science, RMIT University, Melbourne, VIC 3001, Australia; d Centre for Microscopy, Characterisation and Analysis, The University of Western Australia, Perth, WA 6009, Australia; e Department of Electrical and Computer Engineering, University of Massachusetts, Amherst, MA 01003, USA

## Abstract

The data included in this article provides additional supplementary information on our recent publication describing “Inducing tunable switching behavior in a single memristor” [1]. Analyses of micro/nano-structural and compositional changes induced in a resistive oxide memory during resistive switching are carried out. Chromium doped strontium titanate based resistance change memories are fabricated in a capacitor-like metal-insulator-metal structure and subjected to different biasing conditions to set memory states. Transmission electron microscope based cross-sectional analyses of the memory devices in different memory states are collected and presented.

**Specifications table**

**Table:** 

Subject area	*Electrical Engineering, Material Science*
More specific subject area	*Resistive oxide memories, Interface engineering*
Type of data	*Image (Transmission electron microscopy, electron energy loss spectroscopy, energy-dispersive X-ray spectroscopy)*
How data was acquired	*Cross-sectional lamellae are prepared by focused ion beam (FIB) cuts by using a FEI Scios DualBeam*^*TM*^*system and imaged by using JEOL 2100F TEM system.*
Data format	*Analyzed*
Experimental factors	*Electrical voltages in the range of +5 V to −5 V were applied across the top and bottom electrodes of the memory cells to set them in different memory states, prior to the lamellae preparation.*
Experimental features	*TEM images and spectroscopic data are collected using a <1.5 nm beam spot and dispersion of 0.3 eV per pixel.*
Data source location	RMIT University, Melbourne, VIC 3001, Australia
Data accessibility	*With this article.*

**Value of the data**

• The data will provide a guideline to assess the effects of an applied electric field and associated Joule heating on a complex oxide based resistive memory.
• The data provides significant insights on the micro/nano-structural and compositional changes at metal/oxide interfaces and within the complex oxide during different memory states in a resistive oxide memory.
• The data is expected to be an important resource for designing a dynamic memory system exhibiting multiple resistive switching behaviors.
• The data will serve as a benchmark for realizing theoretical models and simulations of resistive oxide memories.
• The data will serve as a key reference for future research in metal-oxide based resistive memories.

## 1 Data

The data provided here is related to the analyses of morphology and composition of a resistive oxide memory based on an amorphous complex oxide, such as oxygen deficient SrTiO_3-*x*_ (*a*-STO_*x*_) [2], [3], [4], [5], in different memory states, namely – pristine, electroformed, low resistive state and high resistive state.

Transmission electron microscope (TEM), energy-dispersive X-ray spectroscopic (EDS) and electron energy loss spectroscopy (EELS) techniques are used to analyze the morphology and composition of the selectively chromium doped *a*-STO_*x*_ (Cr:*a*-STO_*x*_) resistive memory.

### 1.1 Cross-sectional analyses of pristine Cr:a-STO_x_ MIM devices

**Fig. 1:**
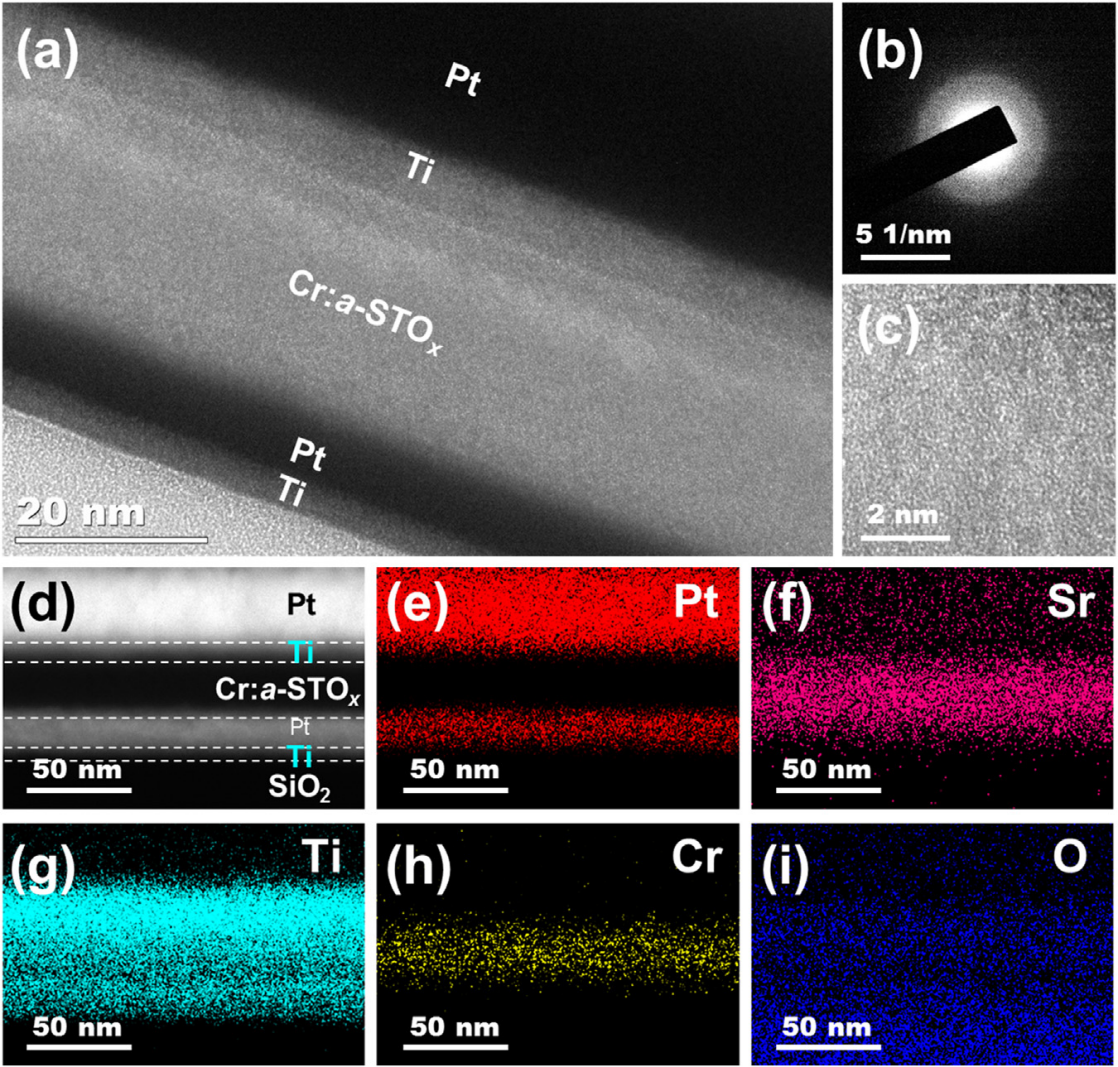
Microstructure structure of the pristine Cr:*a*-STO_*x*_ devices. (a) TEM micrograph of a pristine MIM device. (b) Selected area electron diffraction pattern collected from the MIM cross-section. (c) High resolution TEM micrograph of the Cr:*a*-STO_*x*_ oxide film. (d) High-angle annular dark field TEM micrograph of the pristine MIM device.The elemental EDS mapping of (e) Pt, (f) Sr, (g) Ti, (h) Cr and (i) O.

The cross-sectional TEM and EDS analyses of a pristine Cr:*a*-STO_*x*_ MIM device is presented in Fig. 1. The TEM micrograph (Fig. 1a) of the MIM structure shows the Cr:*a*-STO_*x*_ oxide film is sandwiched between the top Pt/Ti and bottom Pt electrodes. The selected-area electron diffraction (SAED) pattern (Fig. 1b) and high-resolution TEM (HRTEM) micrograph (Fig. 1c), collected from the MIM cross-section show an amorphous structure of the Cr:*a*-STO_*x*_ oxide film. Fig. 1d shows a high-angle annular dark field (HAADF) micrograph of the pristine cross-section in scanning TEM. Fig. 1e-i present elemental EDS maps of Pt, Sr, Ti, Cr and O, respectively. The EDS maps confirm successful Cr doping in the *a*-STO_*x*_ (*via* co-suputtering of Cr and SrTiO_3_) and also the desired MIM structure of the memristive devices to execute multiple resistive switching behaviors.

**Fig. 2:**
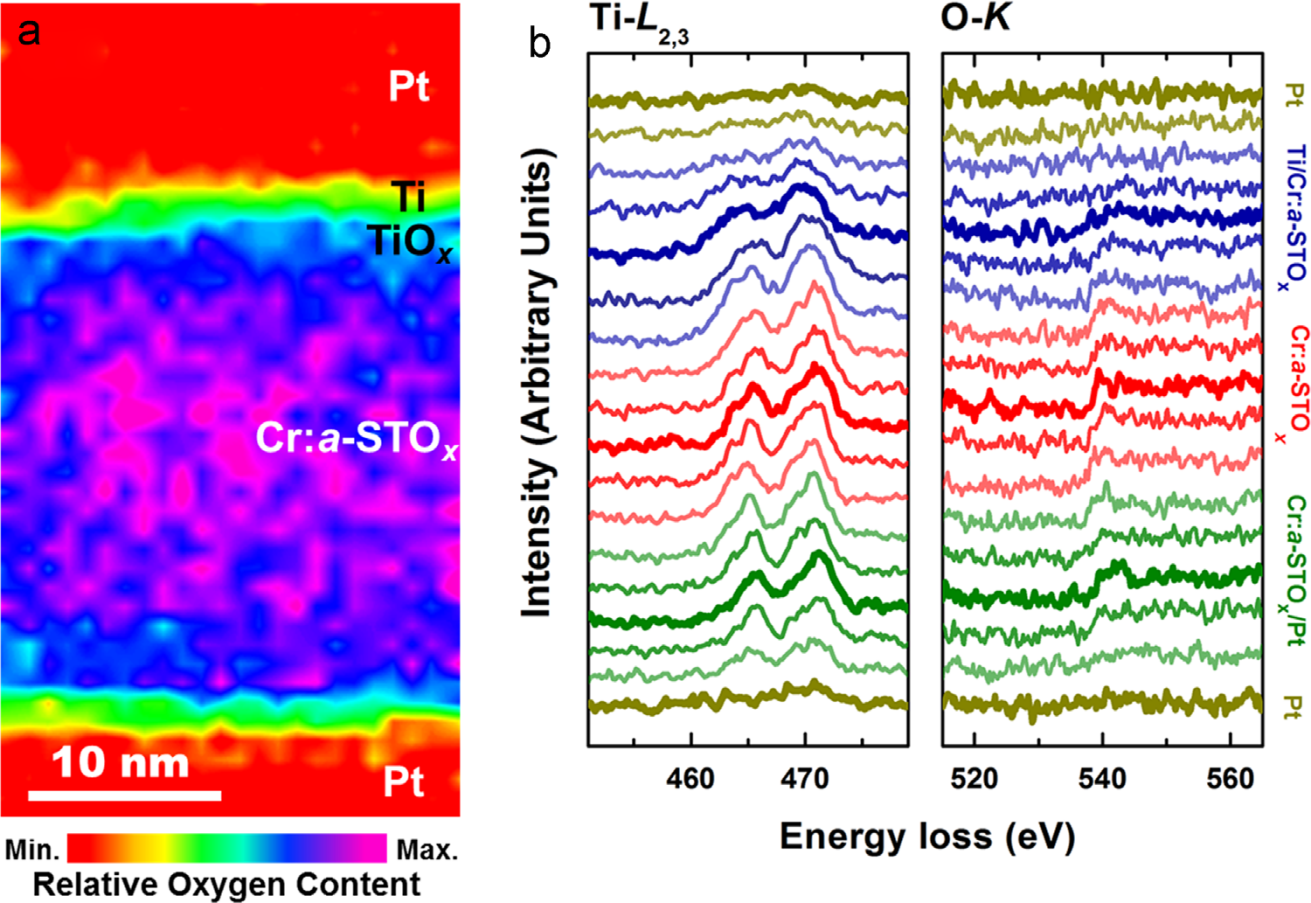
Electronic structure of the pristine Cr:*a*-STO_*x*_ devices. (a) The EELS O–*K* edge area map and (b) the EELS Ti–*L*_2,3_ and O–*K* edge profiles along a line scan across the MIM device.

The electronic composition and the relative distribution of oxygen content across the pristine MIM device is presented in the EELS area map (Fig. 2a), collected by considering the O–*K* edge intensities. The EELS Ti–*L*_2,3_ and O–*K* edge profiles (Fig. 2b) are also obtained from a line-scan across the MIM structure. Broad Ti–*L*_3_ and Ti–*L*_2_ peaks at the top Ti/Cr:*a*-STO_*x*_ interface can be used to identify the oxidation states of Ti (*i.e.*, presence of mixed Ti^2+^ and Ti^3+^ oxidation states at the top interface) as explained in Ref. [6].

### 1.2 Cross-sectional analyses of electroformed Cr:*a*-STO_x_ MIM devices

**Fig. 3:**
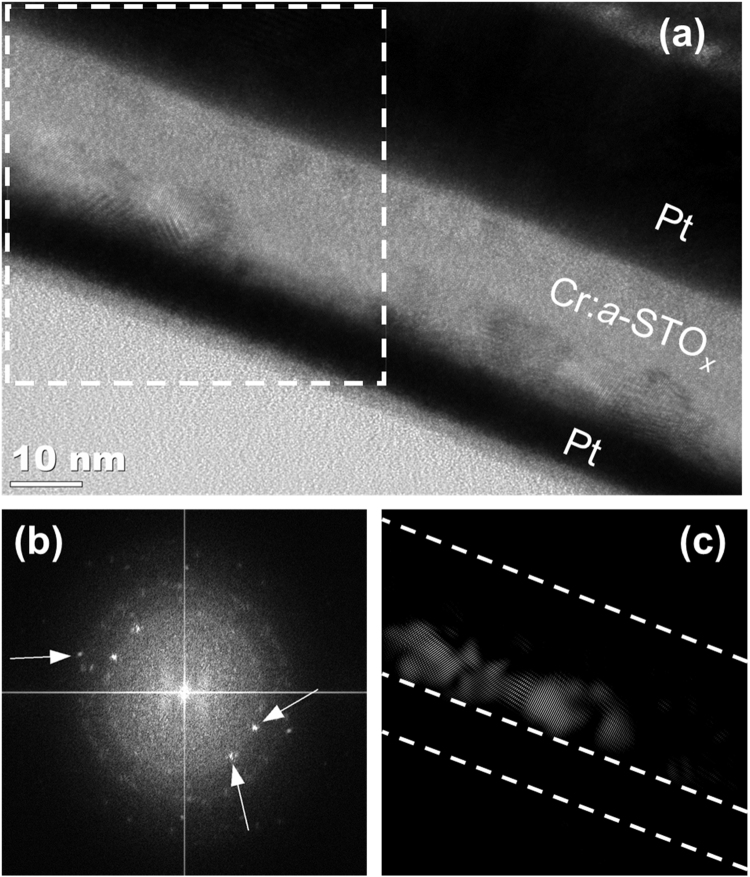
Morphological analyses of the Cr:*a*-STO_*x*_ MIM devices electroformed to exhibit CW-BP resistive switching behavior. (a) TEM micrograph of the MIM device subjected to electroforming step. The box encloses the ROI. (b) The FFT diffraction patterns generated from the ROI enclosed in (a). (c) The iFFT obtained from a diffraction spot in (b) highlight the crystalline region along the bottom electrode.

The electroforming of Cr:*a*-STO_x_ MIM devices is carried out by applying a negative voltage (<−5 V) on the bottom Pt electrode to exhibit clockwise bipolar (CW-BP) resistive switching behavior, prior to the TEM lamella preparation. The highlighted region of interest (ROI, enclosed in a box in Fig. 3a) presents an example of isolated incomplete filaments along the bottom interface. The fast Fourier transform (FFT, Fig. 3b) taken from the ROI presents the diffraction spots. The diffraction spots indicated with arrows can be indexed to the cubic phase of STO and are masked to perform an inverse FFT (iFFT), as shown in Fig. 3c. Several diffraction spots are also used to ensure the iFFT show the distribution of crystals with differing orientations.

**Fig. 4:**
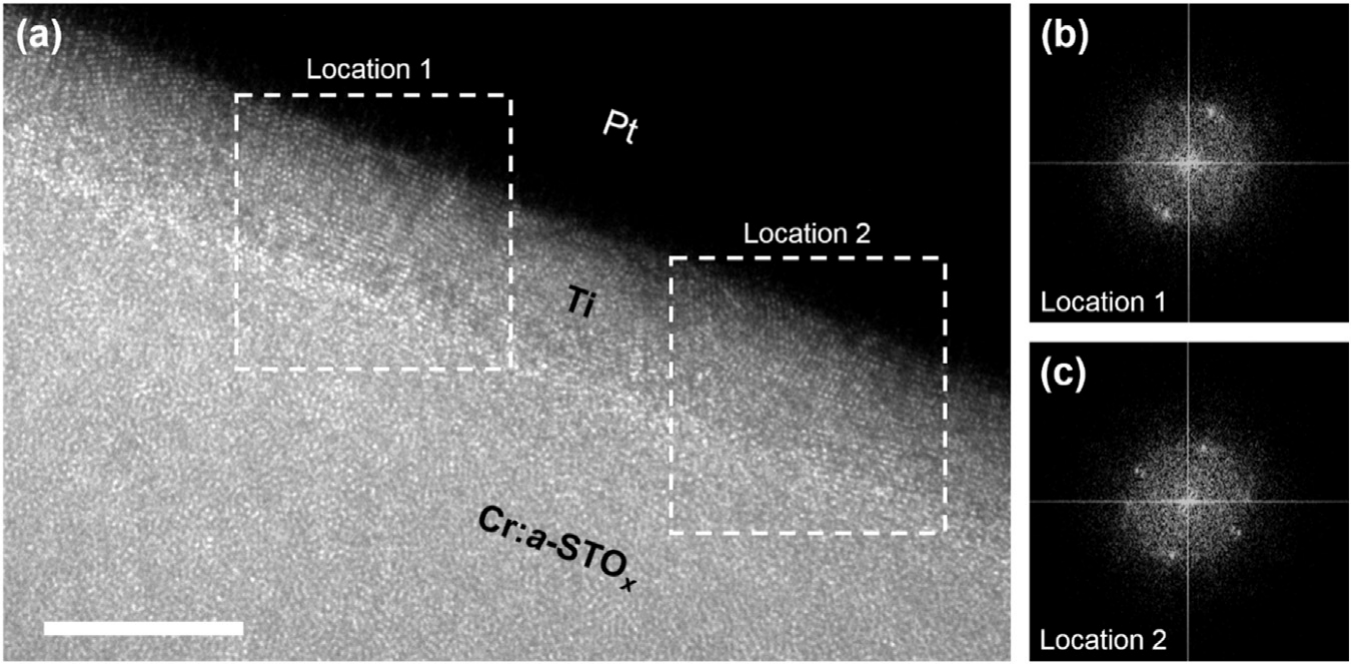
Microstructure of top Pt/Ti/Cr:*a*-STO_*x*_ interface of electroformed MIM devices. (a) TEM micrograph of top interface. Two ROIs are selected at Location 1 and Location 2, enclosed in boxes. Scale bar denotes 10 nm. (b) and (c) are the FFT diffraction patterns generated from Location 1 and Location 2, respectively, in (a).

Fig. 4 presents the cross-sectional HR-TEM micrograph of the top Ti/Cr:*a*-STO_*x*_ interface of a MIM device subjected to the electroforming. In order to assess the morphological changes in the top Ti layer, two ROIs are selected along its length at two different locations (Fig. 4a). The FFT diffraction patterns generated for each location (Fig. 4b,c) show the polycrystalline structure of the top Ti layer. The high intensity diffraction patterns with the *d-*spacing ranging from 2.4 Å to 2.6 Å can be indexed to the different planes of the rhombohedral Ti_2_O_3_.

### 1.3 Cross-sectional analyses of switching Cr:*a*-STO_x_ MIM devices

**Fig. 5:**
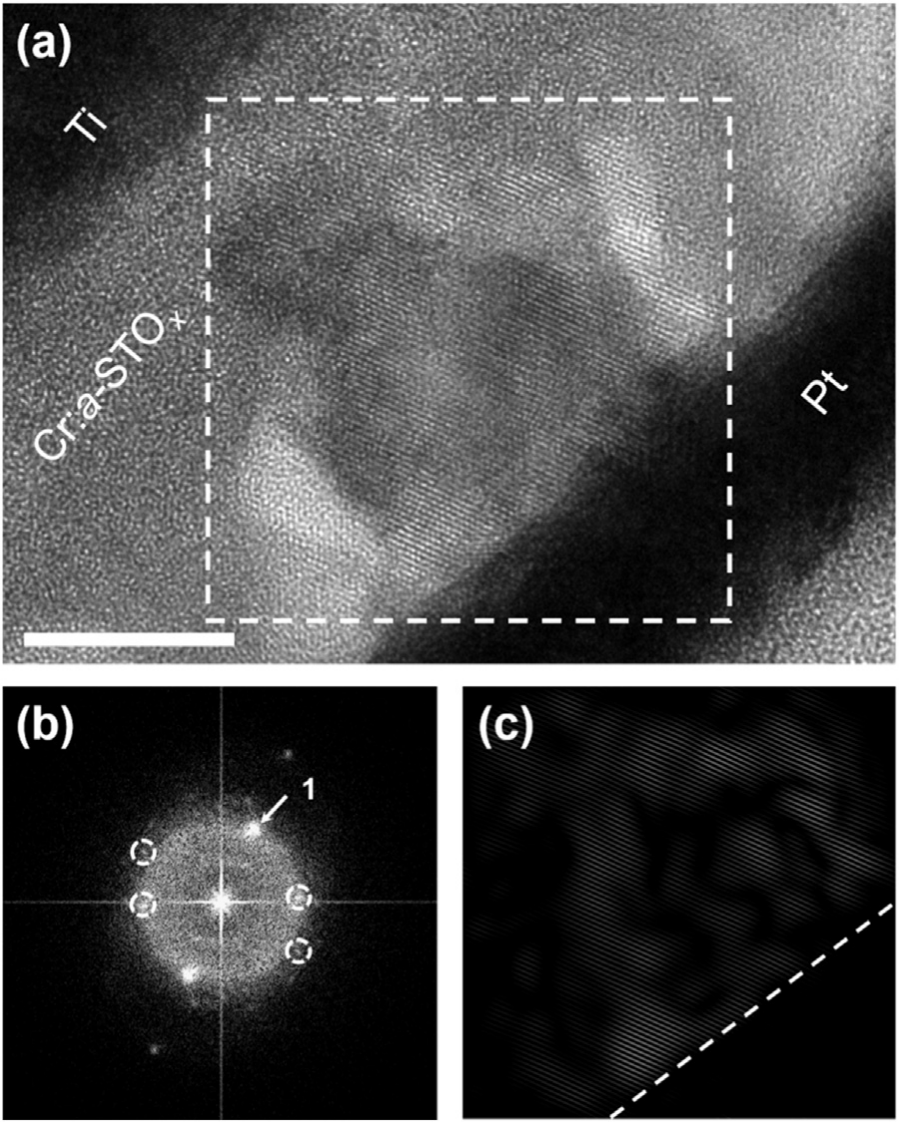
Morphological analyses of the Cr:*a*-STO_*x*_ MIM devices in HRS and exhibiting CW-BP resistive switching behavior. (a) TEM micrograph of the MIM device subjected to at least 100 resistive switching cycles and set to HRS prior to the lamella preparation. ROI is enclosed in the box. Scale bars 5 nm. (b) The FFT diffraction patterns generated from the ROI enclosed in (a). (c) The iFFT obtained from spot 1 in (b) highlight the crystalline region.

Fig. 5a shows the cross sectional HRTEM micrograph of a Cr:*a*-STO_*x*_ MIM device in its HRS, exhibiting CW-BP resistive switching characteristics. The ROI shows the localized crystalline region in the active Cr:*a*-STO_*x*_ layer, extending from the bottom Pt electrode. The FFT diffraction pattern of the ROI (Fig. 5b) shows the presence of different crystalline phases of STO. The diffraction spot of the highest intensity (marked as spot 1 in Fig. 5b) and other weaker diffraction spots can be assigned to the cubic perovskite STO phase. However, the encircled diffraction spots could not be assigned to the cubic perovskite STO phase. The spot 1 (with the *d*-spacing of 2.8 Å) is used to generate the iFFT (Fig. 5c) highlighting the presence of [011] cubic STO phase in the selected ROI.

**Fig. 6:**
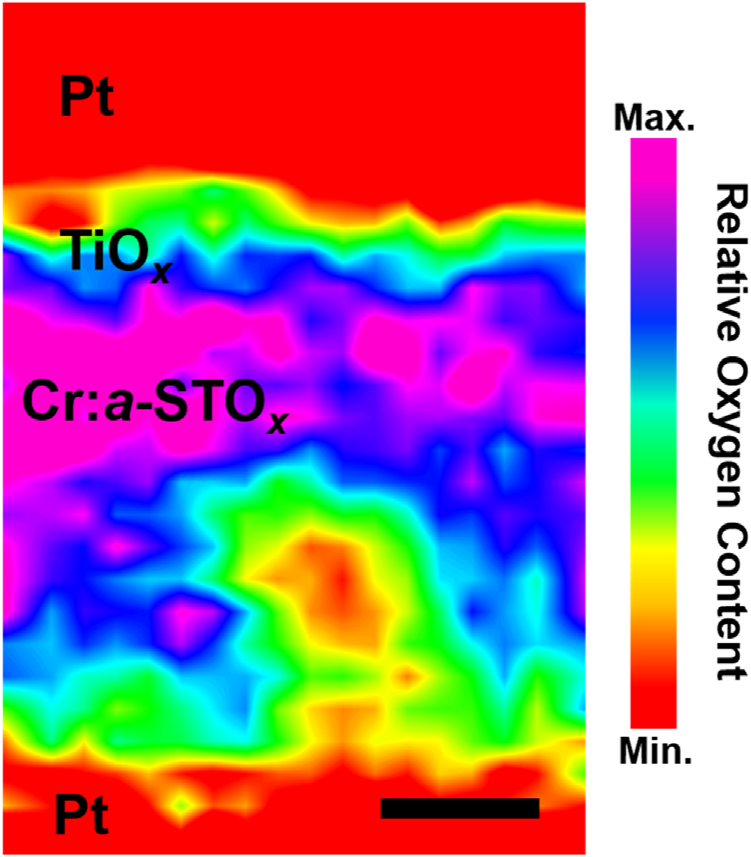
The EELS O–*K* edge area map of the raptured conductive filamentary path in HRS. Scale bar represents 20 nm.

Fig. 6 shows the EELS O–*K* edge area map of the locally crystalline ROI from the MIM device exhibiting CW-BP resistive switching behavior (presented in Fig. 5). The representative MIM device is set to HRS prior to TEM sample preparation. Relatively low oxygen content at the bottom Pt electrode shows a ruptured filamentary path and accumulation of the V_o_s at anode in HRS. Presence of varying oxygen content at the vicinity of top Pt electrode shows the oxidation of Ti layer to a sub-stoichiometric Ti_2_O_3_, as indexed in the FFT analysis (as presented in Fig. 4).

## 2 Experimental design, materials and methods

The Cr:*a*-STO_*x*_ based memory cells are fabricated in metal-insulator-metal (MIM) configuration by following the fabrication steps in Ref. [1], [2], [5]. In the MIM structure Pt (35 nm)/Ti (8 nm) serves as a top metal electrode, Cr:*a*-STO_*x*_ (25 nm) as an insulator and Pt (7 nm)/Ti (3 nm) as a bottom metal electrode. In order to ascertain the effect of applied bias (during electroforming and resistive switching) on the metal/oxide interfaces and within the functional oxide (Cr:*a*-STO_*x*_), the cross-sectional TEM lamellae are prepared *via* FIB cuts from separate MIM memory cells subjected to different biasing conditions namely; pristine, electroformed and switching devices.

## References

[1] Ahmed T. (2018). Inducing tunable switching behavior in a single memristor. Appl. Mater. Today.

[2] Nili H. (2015). Donor-induced performance tuning of amorphous SrTiO_3_ memristive nanodevices: multistate resistive switching and mechanical tunability. Adv. Funct. Mater..

[3] Nili H. (2014). Nanoscale resistive switching in amorphous perovskite oxide (a-SrTiO_3_) memristors. Adv. Funct. Mater..

[4] Ahmed T. (2017). Transparent amorphous strontium titanate resistive memories with transient photo-response. Nanoscale.

[5] Nili H. (2016). Microstructure and dynamics of vacancy-induced nanofilamentary switching network in donor doped SrTiO_3−x_ memristors. Nanotechnology.

[6] Li Y. (2016). Nanoscale chemical and valence evolution at the metal/oxide interface: a case study of Ti/SrTiO_3_. Adv. Mater. Interface.

